# Reducing stigma toward autistic peers: a pilot investigation of a virtual autism acceptance program for children

**DOI:** 10.3389/fpsyt.2023.1241487

**Published:** 2023-11-08

**Authors:** Denise Davidson, Dakota Morales

**Affiliations:** Department of Psychology, Loyola University Chicago, Chicago, IL, United States

**Keywords:** autism, autism acceptance, stigma, remote learning, typically developing children, inclusive education, elementary school

## Abstract

Inclusive educational practices can be beneficial for autistic children, especially when the general education classroom can better meet the child’s academic and socio-emotional needs than a special education classroom. Unfortunately, autistic children may not thrive in general education classrooms if they are perceived negatively, subject to bullying, and are socially isolated and rejected by their typically developing peers. Autism acceptance programs may help address the root cause of these problems, autism stigma. Thus, this study evaluated the effectiveness of a virtual autism acceptance program presented to typically developing, 8–10-year-old children through remote learning technology. The 5-week, stakeholder-approved pilot program included a themed module each week (e.g., facts about autism and reducing stigma, sensory sensitivities, strengths of those with autism) presented through a variety of online educational materials. Pretest, posttest, and maintenance results showed that the program was effective in improving children’s knowledge about autism, and children’s attitudes and behavioral intentions toward their peers with autism. In addition to reducing autism stigma, study findings suggest that remote learning and virtual tools can be used to implement an efficacious autism acceptance program to children, allowing for greater and more cost-effective outreach to children and schools.

## Introduction

The prevalence of autism spectrum disorder or ASD, and its broad range of abilities and impairments (1, 2), present unique challenges to educational systems. This is especially true as the number of children with ASD, henceforth identified as autistic children using identity-first language, in general education settings has grown substantially over the past two decades (3–5). In the United States, this increase was due in part to the passing of the Individuals with Disabilities Education Act (6) and the No Child Left Behind Act (7) that mandated that children with disabilities, including autism, receive an education in the least restrictive environment to the maximum extent possible (8). This increase has also been a response to study findings showing that for a significant number of autistic children with average to above average intellectual ability, special education classrooms did not meet their educational or social–emotional needs (9, 10).

### Benefits and costs of general education placement

Significant benefits of inclusive education for autistic children include increased instructional opportunities and the chance to develop age-appropriate and normalized academic skills, expanded opportunities to develop peer relations and enhanced socio-emotional functioning (9, 11, 12). For example, studies have shown that autistic children in general classroom settings exhibit significant improvements in academic achievement (e.g., math, language arts) when compared to their peers in more restrictive, specialized classroom settings (11, 13). Additionally, inclusion in general education classrooms can lead to improved social and emotional functioning in autistic children because it increases children’s opportunities to interact with their typically developing peers and develop social skills in a classroom setting (14). Equally important, typically developing children benefit from having autistic children in the classroom as it can promote better understanding, knowledge, and appreciation of those with autism as well as other children who may be different from them (12, 15). For example, Noggle and Stites (16) followed three typically developing children for 1 year who were in inclusive preschool programs with at least 40% of the children in the classroom with special needs. All three children showed growth in social skills, improved understanding of human variability, and better acceptance of their peers with disabilities. The parents of one child reported that their child talked extensively about spending time with a visually impaired friend and another child learned that it was possible, and enjoyable, to play a game with someone with limited verbal abilities.

Inclusive education may also reduce stigma, including “autism stigma.” According to Link and Phelan (17) seminal model of stigma, the development of stigma derives from the culturally driven detection and labeling of a difference between groups (e.g., non-autistic versus autistic). That is, perceived differences and an unfavorable distinction between groups can lead to negative labeling, social exclusion, and discrimination of a group (18).

Importantly, research on “autism stigma” suggests that contact with autistic people can reduce the stigma associated with autism. In adults, several studies have shown that knowing someone with autism was associated with more positive attitudes toward autism (19, 20), although the quantity and quality of the interactions mattered (18). In children, while inclusive educational practices may promote better attitudes between those with and without autism, inclusive educational practices alone may not be enough to ensure that an autistic child will thrive in a general education classroom (21, 22). In elementary school settings, parent and teacher reports show that autistic children are significantly more likely to be perceived negatively, be bullied through verbal and physical confrontations, and be socially isolated at school than their typically developing classmates [e.g., (23–28)]. It has been suggested that bullying and other inappropriate behaviors occur because autism is a “hidden disability.” Lacking physical differences, typically developing peers may struggle to understand or emphasize with social and behavioral differences, making autistic children more susceptible to social rejection in and out of the classroom (29).

A lack of understanding toward their autistic peers may occur because typically developing children lack knowledge about autism. Studies have shown that typically developing children are often unable to define autism accurately and may hold erroneous beliefs about autism. For example, they may believe that autism is contagious or that all autistic people are the same (30–32). Additionally, they are often unaware of the challenges autism might pose for a child, including social-communicative issues (e.g., difficulty maintaining eye contact, the use of pedantic speech) and the sensory sensitivities associated with autism (8, 30). As Hebron et al. (33) note, behaviors of autistic students are often misconstrued by their peers in the classroom, with either negative attributions made about these behaviors or the belief that the behaviors of autistic students are fully within their control.

Thus, it is theorized that negative peer relationships in the classroom are due to the reciprocal effects of challenges associated with autism (e.g., social communication difficulties) *and* the lack of knowledge and understanding on the part of their typically developing peers that leads to autism stigma. According to the reciprocal effects peer interaction model (REPIM), a lack of understanding about autism, reduced acceptance of differences, and limited opportunities to learn about autism all contribute to bullying and social exclusion of autistic children and devalue the benefits of inclusive education for them (34).

### The case for autism acceptance programs

In line with the REPIM approach, we believe that negative stigma and inappropriate behaviors toward autistic children in general education classrooms can be lessened through an efficacious autism acceptance program. Autism acceptance programs vary in formats and materials [see (21), for a review]. Most include an educational component to increase children’s knowledge about autism and an attitudinal component to address negative beliefs about autism [e.g., (8, 31, 32)]. For example, Campbell et al. (30) found that a single presentation of autism awareness materials led to improvements in 9- and 10-year-old children’s knowledge about autism at posttest and again 1 week later. Their program also led to improvements in children’s attitudes about autism, particularly for those children with little or no knowledge about autism before the start of the session. However, Cremin et al. (21) note that apart from a handful of studies, autism acceptance programs are often plagued by the lack of theoretical grounding, the narrow or brief implementation of educational materials, and the lack of empirical assessment of children’s learning.

Thus, our goal in the present study was to address these limitations through the pilot implementation of a 5-week autism acceptance program that made comprehensive use of educational materials organized into five themed modules. Each module was designed to increase children’s knowledge about autism and improve their attitudes toward autistic peers. According to attitude change theory, increasing knowledge and improving attitudes toward a group not only results in less stigma toward a group but is essential for improving behavioral intentions and ultimately, behaviors toward others (35–37). This is also consistent with theories on stigma that assert that stigma toward a group is due to problems of knowledge (e.g., ignorance), negative attitudes (e.g., stereotypes), and discriminating behaviors (18). Simply put, this pilot program aims to provide a practical application of addressing stigma by increasing autism acceptance from non-autistic peers.

### The present study

The autism acceptance program was created for this study and implemented during the pandemic (Fall, 2020) to typically developing 3rd and 4th grade children. All program materials were approved by stakeholders (i.e., parents of autistic children, autistic adults) and targeted children’s knowledge about autism, and their attitudes and behavioral intentions toward autistic peers. To our knowledge, a completely virtual autism acceptance program, with all materials shared remotely by the facilitators, had not been developed nor assessed prior to this study.

Research questions and aims were as follows:

1. Can a virtual autism acceptance program lead to significant gains in children’s learning about autism, and maintenance of that learning, between pretest, posttest, and maintenance time points? Additionally, is there evidence that participating in the program leads to a reduction of autism stigma by promoting positive attitudes and behavioral intentions toward autistic children? It is expected that participation in the autism acceptance program will lead to increased knowledge about autism and more positive attitudes and behavioral intentions toward autistic children.
2. Do children show learning of the information from each of the themed modules (e.g., facts about autism, strengths of autistic individuals, sensory sensitivities)? It is predicted that children should be able to learn from each of the modules equally well.
3. Do study results show that a virtual autism acceptance program can be implemented successfully and be viewed favorably by children and school staff? That is, does the program show adequate feasibility (e.g., adherence to guidelines, attendance, lack of substantive problems) and acceptability (i.e., favorable perceptions from children and the teacher)? It is expected that the autism acceptance program can be implemented successfully through a virtual platform (Zoom).

## Methods

### Participants

Twenty-three typically developing children (*M_age_* = 8;09, *SD* = 0;08, age range: 8 years; 02 months – 10 years; 07 months,) attending a private elementary school in a large city in the Midwest region of the U.S. participated. Children (18 males; 5 females) were in a hybrid classroom during the Covid-19 pandemic (Fall, 2020). With social distancing and mask policies in place, children at the school had the option of going into the classroom (*n* = 17) or engaging in remote learning from home (*n* = 6). Both 3rd (*n* = 13) and 4th (*n* = 10) grade children were combined in the classroom, given the small size of each grade at the school. In terms of race/ethnicity, approximately 87% were Latino/a, 10% were White or European American and 3% were Asian American or other. There were no significant differences (*p* > 0.05) between students who chose in-person instruction at their school and students who participated in remote home learning in terms of age, race, or gender. All children in the classroom were fluent in English, as reported by the teacher. The teacher of the classroom regularly taught 3rd and 4th grade. She had been an elementary school teacher for 31 years and had taught all grades between pre-kindergarten through fifth grade, with the exception of kindergarten. The teacher was white and identified as cisgender female. She reported that she occasionally taught a classroom with an autistic child, but that none of the children in her current classroom were autistic. The latter point was supported by parent report and confirmed by the school. On the parent permission letter, parents were asked whether their child had been diagnosed with autism or had other special education needs. They were also asked about their children’s exposure to autistic individuals and to describe the contact. According to the parents, none of the children had been previously diagnosed with autism or had other special education needs. Moreover, none of the children in the program had an Individualized Education Plan (IEP). The IEP is a written plan that specifies educational goals and services that a child with a disability requires in order to succeed in school.

Although over half of the children reported that they had heard of the word “autism” (57%) at pretest, none mentioned having any experiences with autistic children. Parent reports included as part of the parent permission letter confirmed children’s responses. When asked about their children’s exposure to autistic children or adults, none of the parents reported that their children knew an autistic child or autistic adult in any immediate capacity (e.g., family member, friend, current or former classmate). We saw no change in the parents’ responses about their children exposure 1 year later in the parent permission letter completed for the maintenance condition.

In the pretest and posttest conditions, *N* = 23 was obtained following the removal of the data from three children who had significant missing data due to absences. Eighty percent (*n* = 18) of the children participated in the maintenance condition. Of those children not participating, two were no longer at the school, two did not turn in a parent permission slip, and one child was absent on the day of testing.

### Materials

#### Autism acceptance program

Our virtual autism acceptance program consisted of five module sessions, each covering a different theme related to autism. The themes were (1) introducing the facts about autism and reducing autism stigma, (2) learning about the strengths of autistic peers, (3) understanding similarities and differences between typically developing and autistic children, (4) exploring the sensory world of autistic children, and (5) promoting kindness and friendship among typically developing and autistic children. These themes were selected with the goal of improving attitudes and behavioral intentions toward autistic peers. Previous research has suggested that educational components such as these can reduce autism stigma (18, 21). Each of the 5 weekly module sessions were approximately 35 min (*SD* = 33–37 min) in length.

Table 1 shows a more detailed description of the five modules, their goals and the activities presented in each module. All program materials and learning formats were implemented virtually and included online educational materials (videos and workbooks) available at https://researchautism.org/education/students-corner/kit-for-kids/#activitya, brief PowerPoint presentations, classroom discussions, interactive activities and public domain videos (e.g., YouTube Kids). Videos matched the theme of the module and included, but were not limited to, one written and narrated by autistic children, another consisting of interviews with autistic children, adolescents and adults, a video dramatization of an autistic child experiencing sensory overload in a shopping mall setting (e.g., bright lights, loud music) and a video showing the calming benefits of a “sensory room” being implemented at an elementary school.

**Table 1:** Themes, activities and videos for each of the five weekly autism acceptance modules.

Module	Goals for module	Worksheet activities/*Kit for Kids (KfK)*	PowerPoint presentations/classroom discussion	*Autism Tuned In Videos*	YouTube videos
**Module 1:** “*Facts about Autism and Reducing Stigma*”	Introductions Overview of the program Present facts about autism	Children completed workbook p. 1–4 (KfK)	Facts about autism Discussion: Common misperceptions about autism.	*“Everyone is Unique”;* *“What Does That Mean?”*	“*Autism Explained*”
**Module 2:** “*Learning about Autism*”	Learn about an autistic peer (Nick) focusing on his strengths	Reading“*What’s up with Nick?*” booklet Workbook “*What’s up with Nick?*”	Discuss strengths of Nick and others with autism	*“What’s Up with Nick?”*	“*A Sibling Story*”
**Module 3:** “*Similarities and Differences*”	Embracing similarities and accepting differences between all children Understanding the challenges of autism	Activity: How are you similar or different from another? Understanding how autistic peers are the same/different	Brief PowerPoint presentation on topic Children share likes and dislikes Discussion: embracing differences between themselves and a friend or family member	“*Differences Are Ok*”; “*Get into the Act*”; “*Tuned in Together*”	
**Module 4:** “*Exploring Our Senses*”	Exploring the sensory world of autistic children Discuss sensory sensitivities	Activity: Children share examples of sensory experiences they like and dislike. Workbook p. 6–8	Brief PowerPoint presentation on topic Discussion: sensory world of autistic children	“*Spring into Action*”; “*Make it Better*”	“*Can you Make it End?*”; “*The Sensory Room: Helping Students with Autism Focus*”
**Module 5:** “*Encouraging Kindness*”	Promote kindness and encourage friendship with autistic peers	How to be a friend activity. Workbook p. 9	Brief PowerPoint presentation on topic. Students share ideas about how to be friends with an autistic peer	“*Let us Be Friends*”	“*Do all autistic people think the same?*”

Information about access to Organization Autism Research (OAR) videos is on their website. All links to YouTube videos available from the first author. KfK: Kit for Kids (2021) materials and workbooks available on OAR website.

All educational materials used in the program were reviewed by 10 unpaid adult stakeholders before implementation. Four stakeholders were recruited from personal connections that included family and friends who are parents of autistic children (*n* = 2) or are autistic adults (*n* = 2). Six stakeholders were recruited from social media accounts (i.e., Instagram) with no personal connection to the researchers. Two were autistic adults and four were parents of autistic children. Stakeholders were sent an email or direct message briefly explaining the autism acceptance program, asking them if they would provide input on the program. If they responded affirmatively, an email was sent with a link to the videos and attachments of the documents used during the modules. Two stakeholders reviewed the materials of one module, with the modules randomly assigned. Stakeholders were asked to provide brief written comments via email and to determine whether any of the materials used in a module (e.g., videos, PowerPoint presentation, interactive activity worksheets) were unacceptable or needed revising. Based on their feedback, minor revisions were made to various documents (e.g., an activity worksheet) and one video was dropped from the program because several felt it did not differ sufficiently from the other videos shown in that module.

### Measures

#### Autism knowledge measure

A paper and pencil “Autism Knowledge” questionnaire was constructed and included five different categories of questioning (see Table 2). This measure was adapted from the Knowledge of Autism scale (KOA) (39), although several changes were made. For example, on the KOA, children are asked true or false whether autistic students “cannot do normal activities that other people can do.” To reduce reliance on true/false questioning and to be more in alignment with our program, this question was changed to read “*What are some things a child with autism might do or feel?*” with five statements following it such as “*A child with autism can be bothered by lights or sounds*.” Children were asked to put a check mark next to those statements that were correct. Statements following each question were either correct or were incorrect foils (e.g., “*A child with autism is usually very funny*.”). In some cases, children were asked whether they agreed or disagreed with a statement (e.g., I do not know what a child with autism can do.”) by placing a check mark next to it if they agreed (see Table 2).

**Table 2:** Percentage of correct responses between pretest, posttest, and maintenance.

Question	Pretest% correct	Posttest % correct	Maintenance % correct	Cochran’s *Q*		*Post hoc*			
				*χ* ^2^	*p*	Pr-Po	Pr-M	Po-M	*η* ^2^
1. General knowledge									
Have you heard of the word autism before?^a^	59%	–	–	1.32	0.250				
Can you tell me what you think autism is…	8%	73%	61%	13.00	0.002	0.562*	0.625**	0.063	0.28
Can you catch autism from another child like a cold?	76%	96%	94%	1.60	0.449	-	-	-	0.03
Do you think children with autism look different?	44%	59%	78%	2.60	0.273	–	–	–	0.05
Are all kids with autism the same?	72%	91%	100%	5.20	0.740	–	–	–	0.11
2. What are some things a child with autism might do?									
…can do well in school	44%	82%	89%	6.89	0.032	0.312	0.375*	0.062	0.15
…can be bothered by lights or sounds	68%	91%	100%	7.60	0.022	0.187	0.312**	0.125	0.17
…may find it hard to talk to you	76%	77%	89%	1.00	0.607	–	–	–	0.02
…may find it hard to make eye contact with you	68%	91%	94%	6.00	0.050	–	–	–	0.13
…I do not know what a child with autism can do	40%	100%	83%	9.25	0.010	0.438*	0.250	−0.187	0.20
3. Please tell me some other things a child with autism might do or be like									
…can be friendly	56%	91%	94%	9.00	0.011	0.375*	0.375*	0.000	0.20
…can be fun to be around	40%	96%	94%	10.89	0.004	0.437**	0.437**	0.000	0.23
…can be mean and difficult to be around	64%	73%	72%	2.00	0.368	–	–	–	0.04
…is usually very funny^b^	76%	36%	22%	10.8	0.005	0.001	−0.562*	−0.562*	0.23
4. What are some strengths or good things about having autism?									
…can often remember details well	20%	82%	72%	15.17	<0.001	0.562**	0.625***	0.063	0.33
…may be honest and direct when he or she speaks to you	52%	64%	72%	6.55	0.038	0.375	0.375	0.000	0.14
…can be smart	40%	82%	83%	9.50	0.009	0.500**.	437*	0.062	0.20
…will know lots of languages besides English^b^	72%	68%	67%	0.600	0.741	–	–	–	0.01
5. Friendships with children with autism									
Can you be friends with someone with autism?	88%	100%	100%	6.00	0.050	–	–	–	0.13
Can a child with autism be a good friend?	48%	96%	83%	11.40	0.003	0.437**	0.500**	0.063	0.25
Would you like to be friends with a child with autism?	76%	100%	100%	8.00	0.018	0.250	0.250*	0.000	0.17

Pr, pretest; Po, posttest; M, maintenance. ^a^This question was only asked during pretest. ^b^Questions were asked as foils. *p < 0.001, **p < 0.002. Bonferroni corrected error rate across all experiment-wise comparisons is *p* < 0.002. η^2^ = eta squared, maximum-corrected measure of effect size (38). For section 5, at pretest and posttest *n* = 22 and *n* = 17 at maintenance.

Additionally, children were given one open-ended question, “*Can you tell me what you think “autism” is in the space below? If you do not know, please tell me that*.” Children’s responses to this question were scored by two independent coders as “correct” or “incorrect.” Children had to give one to two characteristics about autism for their answers to be coded as correct. For example, one fourth-grade child wrote, “They have a hard time with eye contact and sometime rock in class.” Incorrect responses were almost always due to children not answering it or writing, “I do not know.,” although a few children gave incorrect characterizations (e.g., “They are sad.”).

This measure served as a baseline (pretest), posttest and a maintenance measure. Cronbach’s alpha conducted on the data from the Autism Knowledge measure at pretest (*α* = 0.81), posttest (*α* = 0.90) and maintenance (*α* = 0.79) timepoints showed good reliability across the sample.

#### Module assessments

The researchers created five paper and pencil questionnaires in order to assess children’s learning of information from each of the five modules (see Table 3). Questions were following by three to six statements that children had to decide were correct or incorrect. Specifically, children were asked to check all correct responses from an array of correct and incorrect (foils). Cronbach’s alpha on the module assessments data ranged from adequate to good, *α* = 0.77–0.90.

**Table 3:** Assessment of children’s retention from modules 1–5.

Topics and items	% Agree	% Disagree	One-way χ^2^	*p*	Φ
Module 1: challenges associated with autism					
Sometimes makes noises in the classroom	82.4%	17.6%	7.12	0.001	0.65
Likes to sing and dance*	11.8%	88.2%	9.94	0.020	0.76
Needs to take a break in class	82.4%	17.6%	7.12	0.008	0.65
May rock back and forth	63.0%	37.0%	4.12	0.050	0.49
May flap arms	94.1%	5.9%	13.25	<0.001	0.88
May get up and do jumping jacks*	37.0%	63.0%	4.12	0.050	0.65
Likes to hit others*	18.8%	81.0%	6.25	0.010	0.61
Module 2: strengths associated with autism (*n* = 21)					
May speak directly and honestly with you	52.9%	64.7%	0.059	0.810	0.06
Can be a good friend	70.6%	29.4%	4.88	0.050	0.53
Might know a lot on a particular topic	76.5%	23.5%	4.77	0.030	0.53
Can be really mean*	11.8%	88.2%	9.94	0.002	0.76
Can be smart and do well in school	82.4%	17.6%	3.87	0.050	0.48
Might know a lot on a topic	64.3%	35.3%	1.47	0.230	0.29
Module 3: similarities and differences between children					
All children are similar and different from each other	78.0%	23.0%	7.12	0.008	0.63
Children with ASD only have things in common with other children with ASD*	11.1%	88.9%	10.89	0.001	0.78
Children with ASD can be both similar and different to others	66.7%	33.3%	14.22	0.001	0.89
Module 4: sensory experiences associated with autism					
Children with ASD experience sensory input the same as others*	26.0%	74.0%	4.84	0.050	1.18
A child with ASD may get upset by regular sounds.	53.3%	46.7%	2.73	0.100	0.43
Child with ASD can be overly-sensitive to sensory stimuli (e.g., smells, sights)	86.8%	13.3%	8.07	0.010	0.67
To a child with ASD, a smell may be bothersome…	80.0%	20.0%	5.40	0.020	0.67
Normal sounds can be too loud or annoying to a child with ASD	93.3%	6.7%	11.27	0.001	0.75
To a child with ASD, a normal light might be too bright	93.3%	6.7%	11.27	0.001	0.28
A child with ASD may be really bothered by “outdoor voices” when inside	86.7%	13.3%	8.07	0.005	0.63
It would be best to use indoor voices as children with ASD as they would be less bothered…	93.3%	6.7%	11.27	0.001	0.89
It is best to keep your hands to yourself with children with ASD…	93.3%	6.7%	11.27	0.001	0.002
Module 5: what would a good friend do? (*n* = 22)					
Ignore someone who is different*	95%	5%	16.20	<0.001	0.64
Ask someone what they like to do	100%	0%	–	–	–
Listen when someone is talking	95%	5%	16.20	<0.001	0.64
Make a face if someone acts different*	100%	0%	–	–	–

*Items used as foils to assess bias toward agreement in responding. Φ = Phi measure of effect size. Unless otherwise noted, *N* = 23.

#### Feasibility and acceptability measures

A senior-level undergraduate student recorded how many children were in attendance and how often they participated in the activities at each session. She also kept track of technological difficulties and how much time each session took. After each session, the undergraduate student and the facilitators completed a checklist regarding how well they thought the various activities used in that day’s session (module) were carried out. All of these activities comprised the feasibility measure. Acceptability was gauged with 3 to 4 item paper and pencil feedback questionnaires for each module. Children assessed how well they liked different activities during each module and, once completed, the program as a whole. Children were asked to respond using 3-point Likert scales with 1 = *did not like at all*, 2 = *liked somewhat*, and 3 = *liked a lot*. Children were also asked to rate the activities they liked the best and least from the program. Using a 16-item paper and pencil questionnaire, the teacher was asked to provide feedback on the individual modules, different activities of the program, and the program as a whole. She was also given space to provide open-ended feedback.

### Procedure

Prior to the start of the study, all program materials and activities were reviewed and approved by the Institutional Review Board at the authors’ university. Informed written consent from the school principal, classroom teacher, and parents, and verbal assent from the children were also obtained prior to the start of the program. One week before the program began, children were given the baseline assessment (pretest) to assess children’s knowledge and attitudes (stigma) about autism.

Program facilitators (first and second author) were from a psychology department and included a faculty member and a doctoral student. An advanced undergraduate student completed the feasibility questions as described above. All were online at each autism acceptance session. For each of the five module sessions, the following protocol of activities was used: (1) greetings and the collecting of verbal assents, (2) a review of the previous module’s material and introduction of the current module’s theme through brief PowerPoint presentations and interactive activities, (3) playing videos that matched the theme of the module, (4) worksheet activities with follow-up discussions, and (5) a closing review of the module. Within 24 h after the module ended, the teachers were asked to provide the Module Assessment.

Although children were provided with a PowerPoint review of the previous week’s material, children were not given direct feedback on their individual assessments from the modules. That is, the module assessments were not corrected and returned to the children. Moreover, no feedback was given on the pretest or the posttest questions and statements, in order to protect against children simply remembering how they had responded in the past.

The homeroom teacher was present at all sessions, during which time she connected to us via our Zoom link and made sure our program was broadcast to the classroom via a Smartboard (a large, projector-type screen). She also provided the link to the at-home children and their parents so that they could join from home. Due to the COVID-19 pandemic, all program activities were presented online, and hard copies of assessment tools (i.e., module learning and feedback forms) were delivered to a drop box outside the main office of the school because individuals not affiliated with the school were not allowed in-person visits.

Based on the instructions provided by the researchers, the teacher made sure that the children completed a module learning assessment within 24 h after each module was presented. At the same time, children completed a feedback form that gauged how much they liked specific activities (e.g., interactive activities, videos) from the module. After the final session, children completed an overall assessment (posttest) and the feedback form about the program. In the maintenance condition implemented 1 year later, children completed the posttest again and the behavior intention measure. After the initial posttest, children were given a gift bag that included a certificate of completion, school supplies and a book about autism, chosen by each child from a list of award-winning children’s books on autism. Children were also given a small giftbag of school supplies following the maintenance condition.

### Data analytic plan

All data analyses were conducted using IBM SPSS v28 (Chicago, IL). Cochran’s Q tests were used to assess the first research question (Research Aim 1), “Can a virtual autism acceptance program lead to significant gains in children’s learning about autism, and improvement in children’s attitudes toward those with autism, from pretest to posttest and maintenance time points?” Significant findings were followed up with Dunn’s procedure with Bonferroni correction to control for Type 1 error.

To address our second research question examining whether children retained the material from the modules (Research Aim 2), one-way chi-square analyses were performed on children’s responses from the individual module (1–5) assessments. Of interest was whether children were able to identify correct and incorrect statements in the assessment following each module. Finally, we addressed our third research aim and its questions regarding feasibility (i.e., Can a virtual program be implemented successfully?) and acceptability (i.e., Will children and the classroom teacher view the virtual program favorably?). In addition to children’s learning, we also used positive change in attitudes and behavioral intentions as evidence of a reduction of autism stigma and as a way to judge the efficacy of the program.

No significant differences were found between children who connected with us remotely while in the classroom or at home. Therefore, the results reflect the aggregate analyses of responses. At the maintenance time point 1 year later, only 16 children (70%) participated. Two children were no longer at the school, three children were absent on the day of testing, and two children did not return a parent permission slip.

## Results

### Pretest, posttest, and maintenance assessment

In order to assess children’s learning about autism and improvements in attitudes toward their autistic peers from pretest, posttest and maintenance time points (i.e., Research Aim 1), children were asked questions across the five module themes: (1) general knowledge (“*Can you tell me what you think autism is?*,” “*Can you catch autism from another child like a cold?*”), (2) information about what an autistic child might do or be like (e.g., “*An autistic child can do well in school.*”), (3) strengths about autism (e.g., “*Can often remember details well.,”* “*May be honest and direct when he or she speaks to you?*), (4) sensory sensitivities (e.g., “*An autistic child can be bothered by lights or sounds.*”), and (5) developing friendships with children with autism (e.g., “*Can a child with autism be a good friend?*,” “*Would you like to be friends with a child with autism?*”). Table 2 presents the results of these analyses across time points including percentages of correct responses, *p*-values, *post hoc* analyses, and effect sizes.

As shown in Table 2, children exhibited learning of program material by responding more accurately at posttest and maintenance time points than at pretest. Children showed that they learned information from all five modules (see Table 2). However, the greatest increases in children’s learning included general knowledge about autism [e.g., “*Can you tell me what autism is?*,” *χ*^2^(2) = 13.00, *p* = 0.002, 8, 73, 61% pretest, posttest and maintenance percent correct, respectively], strengths about autism [e.g., “*Can often remember details well*,” *χ*^2^(2) = 15.17, *p* < 0.001, 20, 82, 72% pretest, posttest and maintenance percent correct, respectively], and developing friendships with autistic children [e.g., “*Can a child with autism be a good friend?*,” *χ*^2^(2) = 11.40, *p* = 0.003, 48, 96, 83% pretest, posttest and maintenance percent correct, respectively]. Importantly these findings, along with the remaining findings shown in Table 2, provide evidence that children not only learned the material from the modules but also retained what they learned 1 year later.

The open-ended question, “*Can you tell me what you think autism is in the space below?* “was scored by two independent coders. Coders agreed approximately 98% of the time, with disagreement settled with discussion. At pretest, a significant percentage of the children (92%) were not able to answer this question and either did not answer it, or said, “I do not know.” The few children who answered it correctly at pretest, and the significantly greater numbers of children that answered it correctly at posttest and maintenance time points (see Table 2), had to give at least one or two characteristics that may be present in autism in order for their responses to be scored as correct, e.g., “They have a hard time with eye contact and sometime rock in class.” Although none of the children mentioned that it was a “neurodevelopmental disorder” (information that was given in the program), most children were able to recall and give characteristics that were discussed during the program at posttest and maintenance conditions (see Table 2).

### Individual modules assessment

Children’s retention of information from each module (Research Aim 2) is shown in Table 3. Results showed that children displayed learning about the challenges associated with autism (Module 1), the similarities and differences between autistic and typically developing children (Module 2), and the sensory sensitivities associated with autism (Module 4). However, children were less accurate when asked about the specific strengths associated with autism (Module 3).

Also assessed was children’s learning of material on ways for developing friendships (Module 5). However, when assessing children’s learning from this module, we did not focus solely on making friends with autistic children. Instead, children were instructed, through the presentation of the videos and in our discussion, that making friends with autistic and typically developing children required the same skills and understanding (e.g., by getting to know them, by being tolerant of differences). Children’s performance on the assessment of this material showed they retained it. These results are shown in Table 3.

### Feasibility and acceptability

Finally, we were interested in whether a virtual program could be implemented successfully and be viewed favorably by the children and teacher alike (Research Aim 3). Feasibility checklists and recording of issues that occurred during the program showed only minor problems, such as an internet connection that briefly went out but was resumed within a few seconds. Moreover, checklists showed that the facilitators completed all scheduled activities for each module. In terms of acceptability, children reported that they preferred the videos (animated and real person) and interactive worksheets over other activities. However, children viewed the program and all its activities quite favorably (over 94% reported that they “really liked” the program and all of its activities). The teacher also had a quite favorable review of the program and its presentation of material through a variety of learning formats. When asked why she agreed to participate, she wrote, “…*children should know more about autism because of the likelihood of a child with autism being in one of their classrooms at some point.”* Moreover, she wrote, “*As a teacher, it is important for me, as well as all students in the classroom, to have the knowledge and the tools to be able to welcome a child with autism. Unfortunately, I have witnessed situations in which the teacher and the classroom were unprepared and so the child in the classroom did not thrive.*”

## Discussion

Autism stigma that results in bullying and other inappropriate behaviors toward autistic children may occur because autism may be considered a “hidden disability.” Lacking physical differences, peers may struggle to understand or empathize with social and behavioral differences. Moreover, as Turnock et al. (18) suggest, autistic individuals’ “typical” appearance, coupled with autism-related behaviors, may elevate stigma. That is, socioemotional behaviors associated with autism may be taken as examples of social deviance rather than an underlying difference or difficulty. Others suggest that differences in socio-emotional functioning and social communication make autistic children more susceptible to social rejection and increase autism stigma (29). Consistent with the reciprocal effects peer interaction model (REPIM) (34), a lack of understanding about autism, reduced acceptance of differences, and limited opportunities to learn about autism all contribute to autism stigma. Importantly, the factors that produce autism stigma increase the chances that autistic children will experience bullying and social exclusion in their general education classrooms and devalue the benefits of inclusive education for them.

Thus, the overarching goal of the present study was to pilot test a virtual autism acceptance program based on REPIM principles that addresses negative stigma toward autistic children. Of interest was whether participation in the program led to significant gains in children’s learning about autism and maintenance of that learning between pretest, posttest, and maintenance time points. Also examined was improvement in children’s attitudes and behavioral intentions toward autistic children that were taken as evidence of the reduction of autism stigma.

In terms of overall learning, study findings showed that the program resulted in significant improvements in children’s knowledge about, and attitudes toward, peers with autism between pretest and posttest time points. Moreover, children retained much of this information when assessed a year later. Highlights of the findings showed not only increases in children’s general knowledge about autism, but also children’s learning of information regarding the specific strengths of their peers with autism (e.g., can do well in school, can be smart, can remember details well), and the specific challenges associated with autism (e.g., may find it hard to talk to you or make eye contact with you; may experience sensory sensitivities). Previous research with adults has shown that by providing accurate information about the strengths and challenges associated with autism, and by emphasizing that the challenges are often out of the control of individuals, can autism stigma be reduced (18, 40).

Additionally, positive attitudes about, and behavioral intentions toward autistic children (e.g., can be friendly, would make a good friend) showed significant improvement following our virtual autism acceptance program. This corroborates research by Silton and Fogel (41), who showed that typically developing children were more motivated to play with an autistic child, or partner with them in an academic setting, following the viewing of videos that promoted positive attitudes toward autistic children. Finally, children were more likely to reject common misconceptions about autism, such as “autistic children only have things in common with other autistic children” or that “autistic children experience sensory input the same as others” following participation in the program. Thus, the results of our intervention are in line with approaches that autism stigma can be reduced by using educational tools to increase knowledge and attitudes about autism (18).

Although student feedback revealed that children preferred some formats (videos) over others (PowerPoint presentations), the children and the teacher had quite positive views of all presentation formats and the pilot program as a whole. Based on our findings, and consistent with universal design principles for learning, we would assert that successful autism acceptance programs should provide a variety of different learning formats regardless of mode of transmission (virtual, in-person). This enables multiple ways to learn the material and keeps the 5-week program interesting to children. Thus, our findings support slowly emerging evidence that autism acceptance programs are beneficial for reducing autism stigma through improvements in children’s knowledge, attitudes and behavioral intentions toward their autistic peers (21, 30, 32).

### Limitations and future directions

Although remote learning tools allowed us to present our program virtually, most schools in our area during the pandemic were not utilizing a hybrid model (i.e., students in the same class having the option to learn in-person or remotely from home). Thus, access to additional children was not possible. Moreover, this pilot study consisted of one group, with children serving as their own control (i.e., each participating child completed a pretest, posttest, and maintenance assessment of learning). Equally important, no autistic children participated in the study. Additionally, the teacher who allowed us to implement the program in her classroom had been teaching for over 30 years and was very supportive of the program. Thus, the results of this study may not be generalizable to other school settings, classrooms or the general population. In future research, it will be important to assess the efficacy of our virtual program across more classrooms, and to compare those results with in-person presentations of the program.

Additionally, while we took steps to decrease positivity bias in children’s responding (“yes” to statements), or responding in a socially desirable way, we could not completely eliminate the possibility of these biases. However, children’s performance at pretest, and their rejection of positive foils, argue against strong biases in their responding. Moreover, children were not given specific feedback on their responses on any assessments, although a review of the information from the preceding week’s module was included the following week. Outcomes may have differed with specific feedback on assessments. Future research should also examine how participation in the autism acceptance program translates to real-life behaviors toward autistic children. Nevertheless, the preliminary and promising results of this pilot study suggest that behavioral change through autism acceptance programs is possible because positive change in behaviors cannot occur without first accurate knowledge about, and positive attitudes toward, others (37).

## Conclusion

Preliminary findings from our autism acceptance program showed that a virtual program can address a lack of understanding about autism, reduced acceptance of differences, and limited opportunities to learn about autism in classrooms, factors that have all been shown to contribute to negative stigma associated with autism (34). Thus, online programs such as the present one may provide a new means for autism acceptance materials to be made widely available, expanding the number of schools and children that can be reached. Consequently, significant benefits for both typically developing and autistic children are possible through autism acceptance programs, enabling them to successfully navigate inclusive, general education classrooms together.

## Data availability statement

The raw data supporting the conclusions of this article will be made available by the authors, without undue reservation.

## Ethics statement

The studies involving humans were approved by the institutional review board at Loyola University Chicago. The studies were conducted in accordance with the local legislation and institutional requirements. Written informed consent for participation in this study was provided by the participants’ legal guardians/next of kin.

## Author contributions

DD: conceptualization, funding acquisition, methodology, project administration, supervision, formal analysis, writing–original draft, and editing and review of the manuscript. DM: project administration, data analyses, and writing–review and editing. All authors contributed to the article and approved the submitted version.
